# The Effect of Traumatic Experiences of North Korean Adolescent Refugees upon Their Negative Health Perception: Focusing on Multiple Moderating Effect of Problem-Focused versus Social Support-Focused Coping Strategies

**DOI:** 10.3390/ijerph17249484

**Published:** 2020-12-18

**Authors:** Wonjung Ryu

**Affiliations:** The Center for Social Welfare Research, Yonsei University, Seoul 03722, Korea; wjryu514@gmail.com

**Keywords:** NK adolescent refugee, coping strategies, traumatic experience, negative health perception, problem-focused coping, social support-focused coping

## Abstract

The health problems of North Korean (NK) refugees living a new life after surviving the dangers of life and death traumas is an issue that must be taken very seriously. Adolescent refugees may be particularly vulnerable to adverse physical and mental health issues because of major physical, cognitive, and psychosocial developmental changes during adolescence. This study examines the positive roles two active coping strategies—problem-focused coping and social support-focused coping—can play in NK refugee adolescents’ health self-awareness. The analysis found that “social support-focused coping” alleviates the negative relationship between traumatic experience and health perception, acting as a protective factor. Contrary to our prediction, the protective effect of adopting “problem-focused coping” in this study was not verified. The findings suggest that providing interventions for developing appropriate coping strategies help them live healthier, both physically and mentally, in South Korean society.

## 1. Introduction

In most cases, refugees settle in a new country after experiencing long periods of severe suffering and hardship. In this process, many health risks arise. Adolescent refugees may be particularly vulnerable to adverse physical and mental health issues compared to adult refugees, being in an unstable period characterized by major changes in physical, cognitive, and psychosocial development.

**Figure 1:**
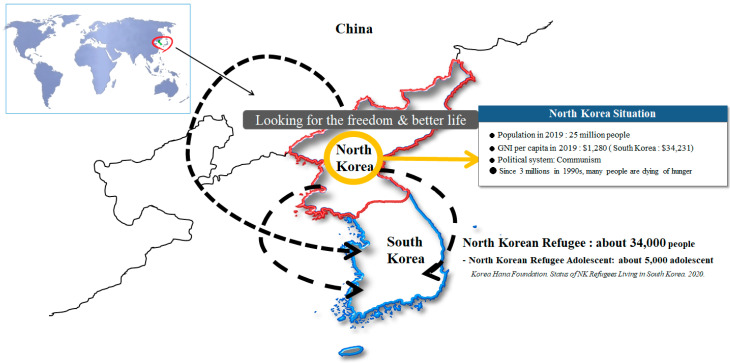
North Korea’s situation and the route of escape from North Korea.

In Korea, there is a group of North Korean (NK) adolescent refugees that can be classified as youth refugees. The Korean Peninsula has not had any civilian exchanges for more than 70 years since it was divided into South Korea and North Korea. Here, “NK refugees” refer to those who enter South Korea, escaping from North Korea’s political persecution or oppression of human rights. As of 2020, the number of NK refugees entering South Korea is estimated at around 34,000, of which about 5000 are adolescents [1]. However, when the number of NK refugee youths currently staying in third countries (China, Mongolia, etc.) while waiting to enter South Korea are included, the estimated range is from at least 7000 to 20,000, see Figure 1. In South Korean society, their health status is relatively poor, and their awareness of their own health status is also worse than South Korean adolescent, which is strongly related to the traumatic incidents they experienced during the events before and after defection [2,3,4,5,6].

A look into their health status indicates that the height and weight attained by NK refugee adolescents are less than those of South Korean adolescents [7]. Specifically, North Korean refugee groups were smaller (0.16 ft to 0.35 ft) and lighter (13.23 lb to 27.56 lb) than South Korean teenagers. This suggests that NK refugee students are significantly weaker, physically, than South Korean students [7] (p. 2).

According to a study by Kim [8] on the health behavior of NK refugees, both the “physical activity level” and “negative health perception level” of NK adolescent refugees were statistically lower than those of South Korean adolescents. As such, NK refugee youths are in a very poor situation not only in their objective health status but also in their subjective perception of their own health. The health problems among NK refugee youths are not just about their physical abilities; they negatively affect body image as well as self-image, causing both serious and minor psychological crises, including depression and anxiety, delinquency behavior, maladjustment, and suicidal thoughts [9,10,11].

As stated at the outset, the health status and subjective health perception of NK refugee youths is highly influenced by traumatic events occurring before and after defection. In general, NK refugees suffer incidents that are difficult for ordinary people to go through once in their lives. In fact, according to a survey conducted in South Korea, 96.5% of NK refugees were found to have experienced traumas before entering South Korea [12]. In a study conducted by Yang [13] on NK refugee youth, 98% of the participants reported that they had experienced trauma more than once. A detailed look into the traumatic incidents experienced by NK refugees are as follows.

First, before they escape from North Korea, NK refugees experience several crises in the course of living in North Korea. In fact, North Korea experienced an economic crisis called “the arduous march” in the 1990s [14]. Following this period, the public distribution system virtually stopped, and food distribution was not possible [15]. To make matters worse, international isolation has continued, referred to as “Songun Politics”, which prioritizes military objectives such as nuclear development or intercontinental ballistic missile development. Combined with these hardships, about two to three million people died or suffered from malnutrition in the mid- to late 1990s [16], and the human rights issues of many children and adolescents being dumped on the streets intensified. In addition, North Korean authorities threaten the health of many NK youth by committing human-rights-related violations such as witnessing public executions, sexual assault, suppression by secret police, imprisonment, and torture [17,18,19], see Figure 1.

It can be said that the traumatic experiences they suffer in the process of defecting after they escape from North Korea are even more serious. As mentioned, youth who escaped from North Korea find their way to South Korea while sheltering in third countries, see Figure 1. In this journey, they cross mountains and rivers, facing the real fear of death, extreme cold and heat, threats from secret police, witnessing the death of others, extreme hunger, forced marriage, and trafficking [20,21]. In addition, after escaping from North Korea, most NK refugees live in seclusion in the border area between China and North Korea for several years, hiding their identities. China, due to diplomatic and political interests with North Korea, is implementing a policy of repatriating NK refugees immediately upon discovery [19] (p. 3). NK refugees repatriated to North Korea in this way are shot or imprisoned in concentration camps.

Furthermore, those who have overcome the fear of death repeatedly, and have successfully entered South Korea, experience another level of trauma in South Korean society. They are subject to social discrimination and exclusion through their status as “refugees” and “thirders”, living in extreme poverty, or being targeted for violence. According to a study by Kim, Kim, Choi, and Nam [22], 10% of NK refugees experience sexual violence in South Korea. Additionally, a study by Um et al. [23] reported that 43.3% of NK refugee participants responded that they had experienced discrimination in South Korean society.

As such, NK refugees experience numerous traumas in the period before and after defection. However, since NK adolescent refugees are less mature than NK adult refugees in physical, emotional, and cognitive terms, the shocks from the traumatic incidents are inevitably greater for them. This problem is often found not only in NK refugee youth but in refugee youth generally. Davidson et al. [24] conducted a research study on refugee children in Australia, reporting that the physical traumatic experiences of refugee children can lead to health problems, such as enuresis and sleep disturbance. In addition, the traumatic experiences of children and adolescent refugees from Afghanistan, Iran, Somalia, Cambodia, etc. were closely related to their own poor general health status [25,26,27,28]. Moreover, according to a study by Yang [13] (p. 2), adolescent refugees tend to perceive and evaluate their physical abilities and health status negatively. Thus, there is no doubt that traumatic experience is an important factor threatening the health of refugee youth, including NK adolescent refugees.

Researchers have been trying to identify protective factors that will alleviate the negative effects of traumatic experience. Through empirical research on various target groups, research has verified protective factors in several dimensions of family variables, school variables, and community variables, such as parental attachment, family intimacy, relationships with peers and teachers, and social support [29,30,31,32]. However, one study focused on “individual variables”, which have been somewhat neglected. Among them, this study specifically focused on “active coping” with stress, because active coping is both an internal factor and a cognitive factor that can lead to a stable protective effect, compared to other factors. Active coping is largely divided into “problem-focused coping” and “social-support-focused coping” [33]. “Problem-focused coping” means a positive attitude for dealing with stressful situations by directly encountering the problematic situations, while “social-support-focused coping” means one asking for another’s help to solve stressful events or situations. The active coping method refers to coping efforts that include facing stress, finding out the cause, and finding necessary resources [34,35]. Although these coping strategies are positively related to individual adaptation, health, and self-care [36,37,38], it is difficult to clearly conclude which coping methods are more effective [39]. This is because the protective effect of the coping method may vary depending on the situations that the study subjects have experienced.

This study aims to examine what positive roles two active coping strategies (problem-focused coping and social support-focused coping) can play in NK refugee adolescents’ health self-awareness (among those who have experienced numerous traumas). This will be a useful resource for identifying effective intervention methods not only for NK youth refugees but also adolescent refugees with health problems around the world.

The research questions being explored based on the above discussion are as follows: (1) what effect does the traumatic experience of NK adolescent refugees have on their subjective health perception? (2) What kind of role does the positive coping method (problem-focused coping/social support-focused coping) play in the relationship between traumatic experiences and health problems?

## 2. Materials and Methods

### 2.1. Sample and Procedures

The subjects of this study are “middle- and high school-age NK refugee youth living in South Korea” identified through a convenience sampling method. Since South Korea and North Korea are still in a political and military conflict, the personal information of NK refugees who have escaped North Korea is highly restricted [40]. Therefore, not only is it extremely difficult to recruit research participants, it is also impossible to obtain a list of the entire population. Thus, it is common to use convenience sampling in studies related to NK refugees. In order to engage participants, this study recruited mainly from middle and high schools and “HANA centers” (which were established to support NK refugees at the national level) in each region. The final analysis data include 202 NK refugee middle and high school students. This research was approved by the institute review board (IRB) of Yonsei University (IRB no. 7001988-201801-HR-184-03).

In addition, because the majority of the participants were minors, the researchers also obtained parental consent forms.

Table 1 shows the general characteristics of the study participants. The sample was comprised of 81 male adolescents (40.1%) and 121 female adolescents (59.9%). Their average age was 17.68 years, ranging from a minimum of 13 years to a maximum of 24 years. The issue of how far the age range of NK adolescent refugees should extend remains open for discussion; many NK refugee students still attend high school even after the age of 20, due to academic blanks in their long escape processes, so this study included those in their 20s as well. Their average duration of stay in South Korea was 62.30 months (SD = 41.46). In response to the question on household income, 88 (43.8%) of the adolescents answered “medium”, 81 (40.3%) of them answered “slightly poor”, and 32 (15.9%) of them answered “wealthy”.

Furthermore, this study consisted of control variables, including a “school maladjustment” variable that can affect the dependent variable, and a “public support services” variable regarding the services they are being provided. The average level of school maladjustment was 2.01 (SD = 0.34, range 1–4); the average level of experiencing public support services was 4.82 (SD = 2.60, range 0–10).

Finally, the results of analyzing the actual conditions of the main variables of this research are as follows: the dependent variable “negative health perception” average was 1.71 (SD = 0.58, range 1–4). “Traumatic experience” was measured with multiple responses: 113 (67.3%) responded that they had experienced trauma in “North Korea”, 68 (40.5%) responded that they had experienced it in “third countries”, and 67 (39.9%) responded that they had experienced it in “South Korea”. “Active coping strategy” was a moderating variable for this study; the average of social support-focused coping was 2.99 (SD = 0.54, range 1–4), which was similar to the average of problem-focused coping—2.97 (SD = 0.48, range 1–4).

### 2.2. Measures

#### 2.2.1. Negative Health Perception

Negative health perception consisted of a single question asking for subjective thoughts about one’s physical condition. The item was rated on a 4-point Likert scale ranging from: “very healthy” (1) to “very unhealthy” (4). The higher the score, the more negative the health perception.

#### 2.2.2. Traumatic Experiences

Traumatic Experiences was measured using the Yoon, Kim, and Han [41] NK traumatic event scale, except for questions for adults. The scale reflects the specific traumatic events NK refugees were likely to experience. The scale consists of 18 items such as forced separation from parents, sexual assault, death of family or close friend, imprisonment, forced repatriation to NK, arrest by police, and so on. Scores were dichotomized as never (0) or at least once (1) and summed. The coefficient that was obtained using Kuder–Richardson’s formula 20 was 0.72.

#### 2.2.3. Active Coping Strategies

The active coping strategies were measured using the Moon and Kim [42] active coping strategies scale. The scale is a revision of the ways of coping (WOC) by Folkman and Lazarus [43], adjusted to suit the situation in Korea.

The active coping strategies consisted of a total of 12 items. Problem-focused coping consisted of 6 items: (1) I knew what had to be done, so I doubled my efforts to make things work. (2) I made a plan of action and followed it. (3) Just concentrated on what I had to do next—the next step. (4) Changed something so things would turn out all right. (5) Drew on my past experiences; I was in a similar position before. (6) Came up with a couple of different solutions to the problem. Social support-focused coping consisted of 6 items: (1) talked to someone to find out more about the situation. (2) Talked to someone who could do something concrete about the problem. (3) I asked a relative or friend I respected for advice. (4) Talked to someone about how I was feeling. (5) Accepted sympathy and understanding from someone. (6) I got professional help.

Each item was measured on a 4-point Likert scale (1 = not at all to 4 = strongly agree). The mean of the questions was used. Cronbach’s α was 0.792 for problem-focused coping, and 0.863 for social support-focused coping strategies.

### 2.3. Data Analysis Plan

Analyses were carried out in SPSS 25.0 (IBM, Armonk, NY, USA) A descriptive analysis was conducted to identify the general characteristics of participants and the conditions of major variables. A multiple regression model was used to test the relationship between traumatic experiences and negative health perceptions, and to verify the moderating effects of active coping (problem-focused coping, social support-focused coping).

## 3. Results

Table 2 presents the results of this study. Model 1 indicates the results of the negative effects of participants’ traumatic experiences on their negative health perception. Model 2 indicates the results of verifying the multiple moderating effect of a problem-focused coping strategy and a social support-focused coping strategy. This model explained about 32.7% of the variance in subjective health perception (*p* < 0.001). The variance inflation factors (VIFs) were examined to check multicollinearity among variables. The VIF score range was from 1.07 to 2.69.

Analyzing the results of model 1 shows that the higher the traumatic experience of NK refugee youths, the higher their negative health perception is. In addition, it demonstrates that among the stress coping strategies, the “problem-focused coping strategy” has a significant negative relationship with their negative health perception. Among the control variables, only school maladaptation was positively associated with negative health perception

In the analysis of model 2, which verified the multiple moderating effect, traumatic experience had a significant effect on negative health perception, as in model 1. The problem-focused coping strategy also demonstrated a negative effect on negative health perception. In the case of the multiple moderating effect, the social support-focused coping strategy verified it as an interaction effect. Specifically, it proves that the social support coping strategy can play a significant role as a “protective factor” for the relationship between traumatic experience and negative health perceptions. On the other hand, the interaction effect of problem-focused coping strategy was not statistically significant.

**Figure 2:**
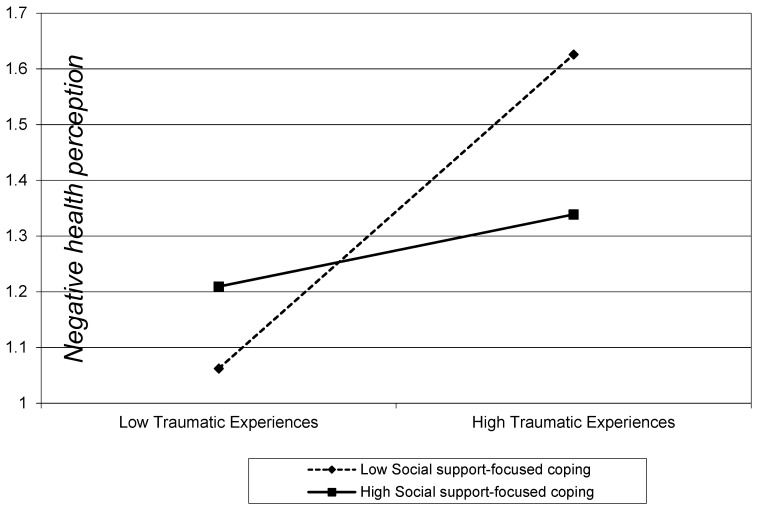
Moderation effect of support-focused coping on the association between traumatic experiences and negative health perception. Range of traumatic experiences and support-focused coping are from (mean –1 SD) to (mean +1 SD). These are the variables before being centered to 0.

Figure 2 is a graph that serves to confirm the moderating effect of the social support coping strategy verified in the above results. The group adopting the social support coping strategy at a higher level has a smaller slope than the group adopting the social support coping strategy at a lower level. This implies that the social support coping strategy functions as a protective factor regulating the relationship between traumatic experience and negative health perception.

## 4. Discussion

The health problems of refugees living a new life after surviving the dangers of life and death traumas are an issue that must be taken very seriously. Refugees of various origins, both domestically and internationally, are exposed to a variety of traumatic events, from the process of escaping from their homeland to the process of settling in a new country, all of which can lead to their own serious health problems [44]. Unlike ordinary migrants, refugees are people who have fled their homelands without prior preparation, so they lack economic, material, and social resources to solve their own health problems. It is very difficult for them to solve their health problems by themselves, so their health difficulties tend to get worse over time [45,46,47]. In particular, adolescent refugees, who are physically and emotionally unstable and immature, may suffer from trauma more seriously than adult refugees.

This study was conducted on NK refugee youths who had escaped from North Korea and settled in South Korea. Currently, South Korea and North Korea are maintaining the Cold War system against each other, and NK adolescent refugees endure a great deal of trauma under this national situation. Human “health” is the basic element for leading a human life that everyone deserves. Therefore, it is very important to help them adapt to South Korean society in good health. Therefore, this study examines how the traumatic incidents experienced in a series of defection processes from North Korea to South Korea affect the subjective health perceptions of NK refugee youths, ultimately aiming to provide specific solutions through verification of protective factors.

First of all, the traumatic experience of NK refugees had a negative effect on subjective health perceptions. This indicates that NK adolescent refugees who were frequently exposed to traumatic experiences have a tendency to negatively evaluate their own health status. According to a study by Yang [13] (p. 2), 98% of NK adolescent refugees reported experiencing at least one traumatic event before and after defection. Compared to such a serious scale, the “treatment services for traumatic incidents and health problems” provided to them in South Korean society are insufficient [13] (p. 2). As soon as NKs enter South Korea, in accordance with the current laws and regulations, they live in a camp for 12 weeks in a national facility called a “HANA center”, receiving education and training to adapt to South Korean society. Basic health check-ups and treatments are performed for them. Nonetheless, this is simply focused on the diagnosis and treatment of their physical diseases, while assessment of their mental health problems caused by traumatic incidents is insufficient. Furthermore, damage caused by a traumatic event may occur immediately after the trauma experience happens, but it often appears gradually after a period of time [48], so in light of the current situation in South Korean society, its medical system to support these people’s health problems can be said to be lacking in the long term [30] (p. 4). Therefore, for their healthy growth, South Korean society must provide long-term medical services based on continuous monitoring of their health problems.

What is more, subjective health perception can be said to be a strong reflection of physical problems as well as psychological factors. As mentioned above, negative evaluation of one’s own physical ability or body image should be treated as important as it can lead to other problems such as maladjustment, delinquency, and suicidal thoughts [49,50]. Hence, so that they do not underestimate and deny their health status for psychological reasons, the nation should adopt support services for mental health problems as well as appropriate interventions in self-esteem and self-efficacy boosting programs, all of which can help them to build positive self-concepts.

Meanwhile, the protective factor that this study focuses on regarded the active coping method for NK refugees to deal with their stress effectively. Thus, this research has demonstrated the role that “problem-focused coping” and “social support-focused coping” can actively play. The analysis showed that “social support-focused coping” alleviates the negative relationship between traumatic experiences and health perceptions. In other words, it verified that the “coping method using social support resources” acts as a protective factor in their subjective health perception. This implies that NK adolescent refugees, who tend to negatively evaluate their health status due to their traumatic experiences, should be offered opportunities to choose a “social support-focused coping” method. Therefore, it also indicates that they need education and training on appropriate stress coping methods, while at the same time they need to have social support resources provided. In order for the “social support-focused coping strategy” to function properly, the basic premise should be that youth refugees are able to open up about their emotions, feelings, and experiences to others. In the case of NK adolescent refugees, they tend to be very alert to others, not easily opening up or exposing their own feelings [51]. Hence, the government and related organizations must identify and provide reliable human resources in order to allow NK refugee youths to comfortably share their grievances. Furthermore, South Korean society should provide opportunities for them to voluntarily express their feelings and experiences at school as well as at home, so negative energy within social networks can be expressed in a healthier way.

Finally, although the protective effect of adopting “problem-focused coping” in this study has not been verified, it is noteworthy that its main effect of alleviating negative health perception has been demonstrated. Similarly, according to the Lee et al. [52], who studied the effectiveness of coping strategies, the two coping strategies (problem-focused coping, social support-focused coping) were interrelated and had a positive effect on the post traumatic growth of youth. This suggests that the effect of problem-focused coping should not be ignored, given the reciprocal association between two coping strategies found in existing studies. Choosing how to cope with stressful events becomes a very important issue for adolescents in order to learn how to function as members of society beyond just health issues. In particular, the process of facing stressful problems such as with the “problem-focused coping strategy” needs to be actively promoted by our society so that it enables adolescent refugees to find a fundamental solution to the situation. Therefore, parents, teachers, and facility workers who closely protect NK adolescent refugees should serve as social supporters, helping the adolescents to be able to identify and resolve the source of the problems together. The intervention measures discussed above will encourage NK adolescent refugees to choose appropriate coping strategies in various crisis situations, ultimately contributing to helping them to live healthier both physically and mentally in such an unfamiliar society.

## 5. Limitations and Suggestions for Future Research

Despite a number of benefits, there are a number of limitations that affect the strength of the conclusions that can be drawn: (1) the subject of the research is not diverse. Since this study was conducted with adolescents who use safely accessible schools and institutions, adolescents outside the protection of schools or facilities were excluded from the study. Adolescents outside the protection of schools or facilities may show more serious health problems, so further studies need to be conducted to include them. (2) The sample size (n = 202) of these data is small. Although this sample size is about 10–12% of the total population, more samples are needed to increase the reliability of the results. Unfortunately, it is not easy for an individual researcher to approach NK refugees in South Korea since their personal information is strongly protected by the government for security reasons. (3) There is a possibility of reduced reporting and social desirability bias for some sensitive variables. Quantitative data in this study are from data that NK refugee adolescents responded to in a self-written manner. Thus, this study has its own limitations in that there is a possibility that they may report differently from the actual levels about school maladjustment, household income, or health awareness questions.

## 6. Conclusions

NK adolescent refugees enter South Korean society with new aspirations. Currently, although the South Korean government and communities are seeking to provide many services and benefits to them, there are many obstacles that refugees must overcome in order to achieve successful settlement in South Korean society. In particular, they need more time and effort to adapt to society due to physical illnesses and emotional problems caused by traumatic experiences. As reviewed in this research, active coping strategies against stress can not only effectively solve the difficulties experienced by NK adolescent refugees who have negative health perceptions due to traumatic experiences but also contribute to the preparation of policy measures within the nation for the increasing population of young NK refugees. Above all, the author argues through this study that continuous efforts and interest are required to aid refugee youths worldwide who are suffering from health issues.

## Figures and Tables

**Table 1:** Sample characteristics (*N* = 202).

Variable		*N* (%)	M (SD) *	Range
Sex	Man	81 (40.1)		
	Woman	121 (59.9)		
Age		-	17.68 (2.53)	13–24
Duration in South Korea (monthly)		-	62.30 (41.46)	1–201
Subjective income	Lower class	81 (40.3)	-	-
	Middle class	88 (43.8)	-	-
	Upper class	32 (15.9)		
School maladjustment			2.01 (0.34)	1–4
Public support services			4.82 (2.60)	0–10
Subjective health perception		-	1.71 (0.58)	1–4
Traumatic experience	North Korea	113 (67.3)	-	
	Third countries	68 (40.5)		
	South Korea	67 (39.9)		
Problem-focused coping		-	2.97 (0.48)	1–4
Social support-focused coping		-	2.99 (0.54)	1–4

* Mean scores of continuous variables before being centered to 0.

**Table 2:** Multivariable regression of negative health perception (*N* = 202).

Variables	Model 1			Model 2		
	B(SE)	*β*	95% CI	B(SE)	*β*	95% CI
Constant	1.413 (0.635)		[0.159, 2.667]	1.309 (0.626)		[0.072, 2.546]
Sex	0.092 (0.087)	0.075	[−0.080, 0.264]	0.086 (0.086)	0.070	[−0.083, 0.256]
Age	0.01 (0.018)	0.042	[−0.026, 0.045]	0.011 (0.018)	0.050	[−0.024, 0.046]
Duration in South Korea	0.001 (0.001)	0.052	[−0.001, 0.003]	0.000 (0.001)	0.017	[−0.002, 0.002]
Subjective income	0.033 (0.039)	0.07	[−0.045, 0.111]	0.050 (0.039)	0.104	[−0.028, 0.127]
School maladjustment	0.493 (0.144)	0.264 **	[0.208, 0.777]	0.478 (0.144)	0.256 **	[0.193, 0.762]
Public support services	−0.016 (0.017)	−0.069	[−0.049, 0.017]	−0.011 (0.017)	−0.050	[−0.045, 0.022]
Traumatic Experiences (A)	0.113 (0.057)	0.173 *	[0.001, 0.225]	0.123 (0.056)	0.189 *	[0.013, 0.233]
Problem-focused coping (B)	−0.323 (0.161)	−0.225 *	[−0.642, −0.005]	−0.350 (0.160)	−0.244 *	[−0.666, −0.033]
Social support-focused coping (C)	−0.107 (0.127)	−0.094	[−0.358, 0.144]	−0.065 (0.127)	−0.058	[−0.317, 0.186]
A × B				0.044 (0.153)	0.031	[−0.258, 0.345]
A × C				−0.262 (0.127)	−0.220 *	[−0.514, −0.010]
R^2^		0.292 ***			0.327 *	
Adjusted R^2^		0.249			0.277	
F		6.908 ***			6.575 ***	

* *p* < 0.05, ** *p* < 0.01, *** *p* < 0.001.
